# The Changing Trend in Clinical Characteristics and Outcomes of Male Patients With Urethral Stricture Over the Past 10 Years in China

**DOI:** 10.3389/fpubh.2021.794451

**Published:** 2021-12-24

**Authors:** Xu Cheng, Mao Ding, Mou Peng, Lizhi Zhou, Yijian Li, Shuang Peng, Shunhua Cheng, Yinhuai Wang

**Affiliations:** ^1^Department of Urology, The Second Xiangya Hospital, Central South University, Changsha, China; ^2^Department of Nephrology, Renmin Hospital of Wuhan University, Wuhan, China

**Keywords:** urethral stricture, characteristic, outcome, retrospective, socio-economic

## Abstract

**Background:** Male urethral stricture is a disease with a high incidence rate. With social-economic development in the developing countries, the trend of etiology and treatment of male urethral stricture changed was speculated.

**Methods:** The clinical data of the male patients with urethral stricture from 2000 to 2019 were analyzed. The subjects were divided into Group A (2000–2009) and Group B (2010–2019) according to treatment time. The pooled analysis of the data extracted from pieces of literature was also performed.

**Results:** About 540 patients were included in the present study, including 235 patients in Group A and 305 patients in Group B. In recent 10 years, trauma has still been the main cause of urethral stricture. Iatrogenic injury, especially transurethral operation, increases significantly, while male urethral stricture secondary to radiotherapy and infection decrease. Urethroplasty increases and the reoperation rate decreases in treating simple urethral stricture, and flap urethroplasty also increases in treating complex urethral stricture. The results of a pooled analysis of data from 11 centers in Mainland China are partially consistent with it. Complications, such as urethral fistula, false canal, ejaculation disorder, and penile curvature, decrease significantly.

**Conclusions:** The main causes of urethral stricture in the recent 10 years are still trauma and iatrogenic injuries, and the etiology of urethral stricture is related to socioeconomic development. With the increase of intracavitary minimally invasive treatment and flap urethroplasty, the curative effect is increasing, while iatrogenic urethral stricture cannot be ignored.

## Introduction

Male urethral stricture (MUS) is a common disease and manifested by dysuria, which is often accompanied by urinary tract infection, and even damages renal function, causes male sexual dysfunction, and lowers the quality of life of patients (1). Due to the unbalanced social-economic development in the various regions of China, under strengthened security measures in working, the incidence of MUS in developing countries is generally higher than that in developed countries (2). As a populous country with developed infrastructures and coal industry, China has suffered a lot from MUS-related social burden, while Hunan Province is a miniature of this situation to some extent. There is no national epidemiology investigation of MUS at present (3); hence, it is necessary to analyze the trend of MUS in various regions of China.

The Center attracts many patients with MUS from the whole province and surrounding areas. The clinical data of patients with MUS were collected in the present study, and the trends of etiology, treatment methods, and outcomes of them were analyzed in the past 20 years, aiming to fill pieces of literature in this field.

## Methods

### Patients

Patients with MUS treated in The Second Xiangya Hospital of Central South University from January 2000 to December 2019 were included. The present study was approved by the Ethics Committee of the Hospital, and informed consent was obtained from each patient. The authors had no access to information that could identify individual participants during or after data collection. Inclusion criteria were as follows: (1) ≥18 years old; (2) patients with a history of MUS; (3) patients with symptomatic MUS diagnosed by urethroscopy and urethrography; (4) patients who received treatment related to MUS, but failed to recover; (5) patients with external urethral orifice stricture, and diagnosed with lichen sclerosus. Exclusion criteria were as follows: (1) patients with MUS caused by malignant tumors; (2) patients with untreated hypospadias; (3) patients diagnosed with MUS, accompanied by acute urinary tract infection for the first time, and only received suprapubic cystostomy and anti-infective therapy.

### Study Design

The present study was a single-center, retrospective, and observational study. The included patients were assigned into two groups according to the primary visitation date: Group A (January 2000–December 2009) and Group B (January 2010–December 2019). This study was reported according to the *Strengthening the Reporting of Observational Studies* in Epidemiology Guidelines for Cohort Studies.

### Measurements and Evaluation

The clinical data of basic characteristics, stricture site, etiology, comorbidities and treatment strategies, and one-year follow-up were collected.

Criteria for effective treatment: within 1 year after the urethral catheter was removed; the diameter of the urethra was ≥10 F under cystoscopy. The patients were satisfied with excretion of urination and experienced no obvious dysuria or dripping. Furthermore, the International Prostate Symptoms Score (IPSS) was ≤ 12, the Quality of Life (QoL) score was ≤ 3, and the urodynamic test Qmax was ≥15 ml/s. No distinction was made on whether self-catheterization or urethral dilatation was performed in the analysis. Definition of a complex MUS is given as follows: (1) posterior MUS of >2 cm, surrounded by scars caused by pelvic floor hematoma muscularization; (2) accompanied by diverticulum, false tract, and fistulous tract, regardless of the length of the MUS; (3) accompanied by urethral sphincter injury (4).

The clinical data were collected by two researchers independently and submitted to a third researcher for verification. Conflicts in the data were further verified by a third researcher. In addition, the number of patients with missing follow-up data was controlled strictly. When the number of patients lost to follow-up exceeded 10% of the total 560 patients, the general characteristics of the patients who were lost to follow-up and those who were not lost to follow-up were compared.

### Pooled Analysis

In order to make the sample more representative and make further comparisons from different aspects, multicenter data were included in the study. China was a developing country with uneven economic and social development. The pooled analysis was based on two consensuses: (1) the social economy is developing over time no matter in which region of China; (2) in the same time, the social economic level gradually increased from the northwest to the southeast in China, and this trend has not changed in the past 20 years. We controlled one of the variables in each step of pooled analysis. A literature retrieval was performed in March 2021 using the China National Knowledge Infrastructure databases. Studies on MUS conducted in different periods and in areas with different economic development levels were selected, and then clinical data were partially extracted and added to our data for pooled analysis. The analysis process included two parts: first, the patients were grouped by treatment time, and then the comparison of the MUS site, etiology, and treatment options was conducted so as to reveal the trend of the disease in China over time; second, the patients were divided into two groups according to the economic level of the area where the hospital is located, and then the same comparison was performed so as to reveal the trend of the disease with different economic levels in Mainland China.

### Statistical Analysis

All data were processed with SPSS 22.0 Software, and a two-sided *p* < 0.05 was defined as statistically significant. If the data followed a normal distribution and showed equal variance, Student's *T*-test was performed; when the data did not show a normal distribution or homoscedasticity, the Mann–Whitney U test was performed. The mean and SD are expressed as *x* ±*s*. Qualitative data were analyzed using Pearson's χ2 test (*N* ≥ 40 and *T* ≥ 5), the continuity correction χ^2^ test (*N* ≥ 40 and 1 ≤ *T* < 5), or Fisher's exact test (*n* < 40 or <1).

## Results

### Baseline Characteristics

A total of 540 patients were included in the present study. There were 235 patients in Group A (January 2000–December 2009), and the average age of them was 52.79 ± 14.40 years old. There were 305 patients in Group B (January 2010–December 2019), and the average age was 53.18 ± 15.35 years. There was no significant difference in the length and the site of the urethral stricture between these two groups. The follow-up data were missing in 16 patients in Group A and 17 patients in Group B. This was <10% of the sample size.

The most important cause of urethral stricture for the patients in Group A and Group B was trauma, with a proportion of 41.70 and 47.21%, respectively. Iatrogenic urethral strictures ranked second, with a proportion of 34.04 and 31.80%, respectively. Among these, the urethral stricture caused by transurethral procedures significantly increased (11.49 vs. 18.69%, *P* = 0.022), while the urethral stricture caused by pelvic radiotherapy and urinary tract infection significantly decreased (7.23 vs. 3.28%, *P* = 0.037; 13.20 vs. 7.87%, *P* = 0.043) (Table 1).

**Table 1:** Baseline characteristics of the male patients with urethral stricture.

**Characteristic**	**GroupA** **(*n =* 235)**	**GroupB** **(*n =* 305)**	***t/** **χ** ^**2**^*	***P*** **-Value**
**Age (yr.)**	52.79 ± 14.40	53.18 ± 15.35	0.300	0.762
**Length of UR**			1.149	0.563
<2 cm	127	163		
2–5 cm	79	95		
>5 cm	29	47		
**UR site**			0.979	0.964
Urethral orifice	21	25		
Penile urethra	36	45		
Bulbar urethra	54	75		
Membranous urethra	85	117		
Prostatic urethra	26	29		
Multiple sites	13	14		
**Trauma**	98	144	1.630	0.202
Pelvic fracture	69	102	1.022	0.312
Riding injury	29	42	0.238	0.626
**Medical injury**	80	97	0.302	0.583
Transurethral procedure	27	57	5.237	0.022
Hypospadias surgery	19	23	0.055	0.815
Indwelling catheter	8	10	0.006	0.936
Instillation of bladder	9	7	1.087	0.297
Radiotherapy	17	10	4.372	0.037
**Urethra infection**	31	24	4.111	0.043
**Lichen sclerosus**	12	13	0.214	0.644
**Unknown causes**	14	17	0.036	0.849

*UR, urethral stricture*.

### Treatment Strategy

Among the patients with simple urethral strictures, 65.00% of the patients in Group A received blind metal urethral sound dilatation, 22.50% of them received endoscopic surgery, and 12.5% of them received urethroplasty; while 63.57% of the patients in Group B received endoscopic surgery, 39.29% of them received direct visual balloon dilatation, and 24.29% of them received direct visual internal urethrotomy. Furthermore, in Group B, the number of patients who received blind metal urethral sound dilatation significantly decreased (*P* = 0.000), while the number of patients who received direct visual balloon dilatation significantly increased (*P* = 0.000) (Table 2).

**Table 2:** Treatment options between the two groups.

**Treatments**	**Simple urethral stricture**			**Complex urethral stricture**		
	**Group A (*n =* 80)**	**Group B(*n =* 140)**	***P*** **-Value**	**Group A (*n =* 155)**	**Group B (*n =* 165)**	***P*** **-Value**
**MUSD**	52	20	0.000			
**Endoscopic surgery**	18	89	0.000	30	24	0.019
DVBD	1	55	0.000			
DVIU	17	34	0.608	30	24	0.019
**Anastomotic urethroplasty**	8	23	0.187	75	49	0.001
SRPA	8	23	0.187	42	28	0.029
PUPI				33	21	0.041
**PFU**	2	7	0.368	46	96	0.000
foreskin-PFU	2	7	0.368	7	28	0.000
scrotal-PFU				39	68	0.002

*MUSD, mental urethral sound dilatation; DVBD, direct visual balloon dilatation; DVIU, direct visual internal urethrotomy; SRPA, stenosis resection plus anastomosis; PUPI, posterior urethral pull-in; PFU, pedicle flap urethroplasty*.

For the patients with complex urethral strictures, compared with Group A, the patients who received urethroplasty (including anastomotic urethroplasty and flap urethroplasty) in Group B increased to 87.88%, especially with flap urethroplasty (*P* = 0.000), while the patients who received direct visual internal urethrotomy decreased to 14.55% (*P* = 0.019) (Table 2).

### Treatment Effects

This part of the results is shown in Table 3. For the patients with simple urethral strictures, the overall operation success rate was 84.21 and 91.60% in Groups A and B, respectively (*P* = 0.103), and the reoperation rate within 1 year decreased from 53.95 to 31.30% (*P* = 0.011). At the same time, the times of dilations and regular urethral dilatation periods in Group B significantly improved, when compared with Group A (*P* = 0.000, *P* = 0.000) (Table 3). For the patients with complex urethral strictures, compared with Group A, the overall operation success rate was 65.73 and 67.51% in Groups A and B, respectively, (*P* = 0.181), and the reoperation rate within 1 year decreased from 27.27 to 14.01% (*P* = 0.004). The reoperation methods mainly included open surgery and direct visual internal urethrotomy.

**Table 3:** Therapeutic outcomes between the two groups.

**Outcomes**	**Simple urethral stricture**			**Complex urethral stricture**		
	**Group A (*n =* 76)**	**Group B(*n =* 131)**	***P*** **-value**	**Group A (*n =* 143)**	**Group B (*n =* 157)**	***P*** **-value**
**Overall success** ^a^	64	120	0.103	94	106	0.181
**Regular dilatation**	55	63	0.001			
**Dilatation Times (number)**	15.78 ± 3.14	11.50 ± 2.49	0.000			
**Dilatation period (week)**	3.13 ± 1.36	4.49 ± 2.01	0.000			
**Readmission for operation**	41	47	0.011	39	22	0.004
DVIU				27	12	
Open surgery				12	10	
**Adverse events**						
Gross hematuria	28	31	0.043	21	13	0.080
^b^Urinary diversion	14	10	0.019	4	2	0.430
UTI	12	16	0.468	14	15	0.945
Ejaculation disorder	0	1	0.633	17	8	0.033
Penile curvature	0	0		20	11	0.047
Urethral calculi	1	0	0.367	7	9	0.747
Erectile dysfunction	5	8	1.000	27	29	0.928

a *Overall success means no < 1 successful operation within 1 year; DVIU, direct visual internal urethrotomy;*

b *Urinary diversion indicates false passage or urinary fistula; UTI, urinary tract infection*.

Postoperative complications are listed in Table 3. For the patients with simple urethral strictures, compared with Group A, the proportion of gross hematuria in Group B decreased from 36.84 to 23.66% (*P* = 0.043), and the proportion of urinary fistula or false tract in Group B decreased from 18.42 to 7.63% (*P* = 0.019). For the patients with complex urethral stricture, compared with Group A, the proportion of penile curvature after surgery in Group B decreased from 13.99 to 7.01% (*P* = 0.047), and the proportion of ejaculation disorder in Group B decreased from 11.89 to 5.10% (*P* = 0.033). However, there was no significant difference in the remaining complications between these two groups.

### Trends of Disease Characteristics in China

**Figure 1:**
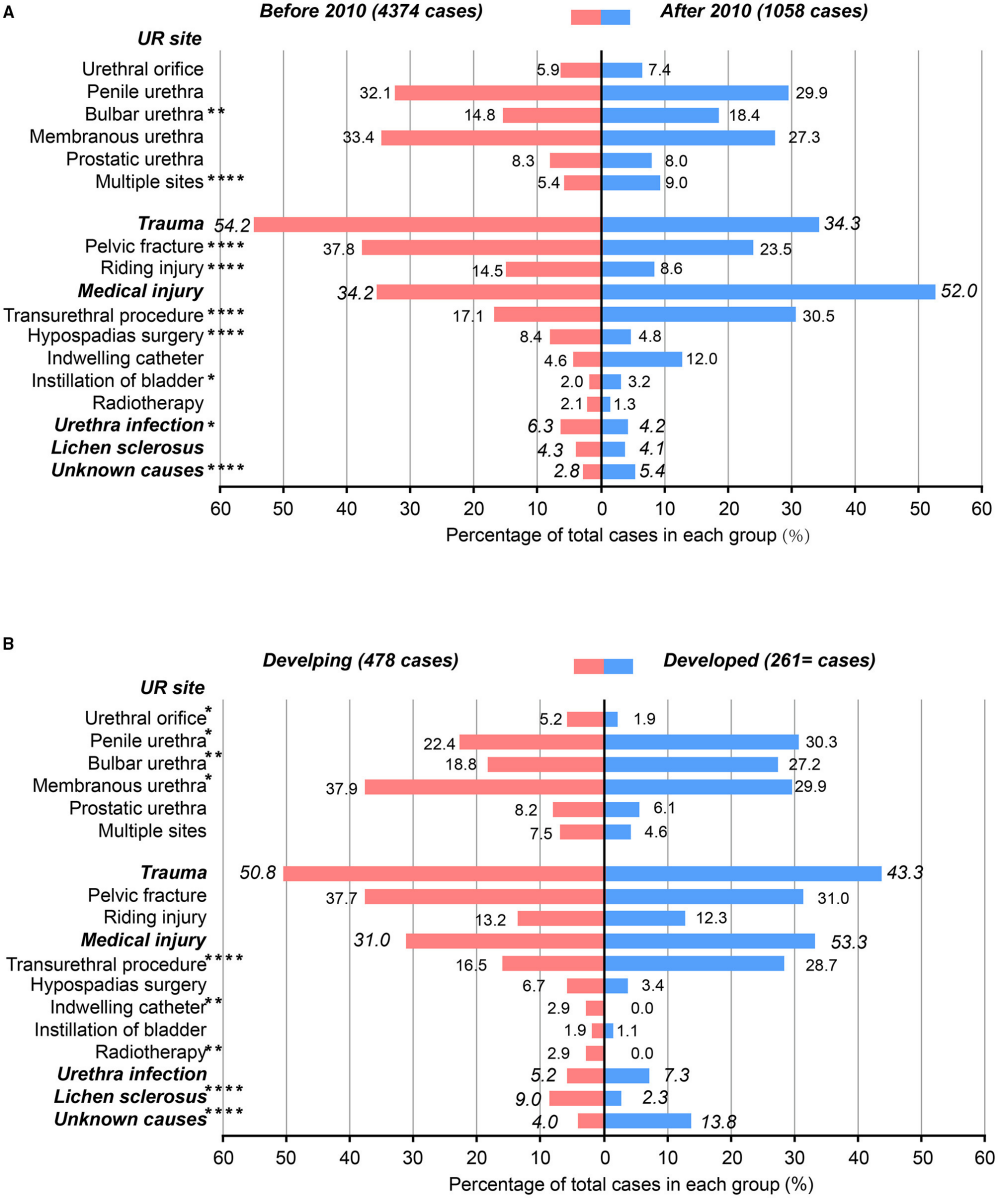
Characteristics of the included male patients with urethral stricture. **(A)** The patients were grouped by treatment time; **(B)** the patients were grouped by developing and developed regions. ^*^ = *p* < 0.05; ^**^ = *p* < 0.01; ^***^ = *p* < 0.001; ^****^ = *p* < 0.0001.

**Figure 2:**
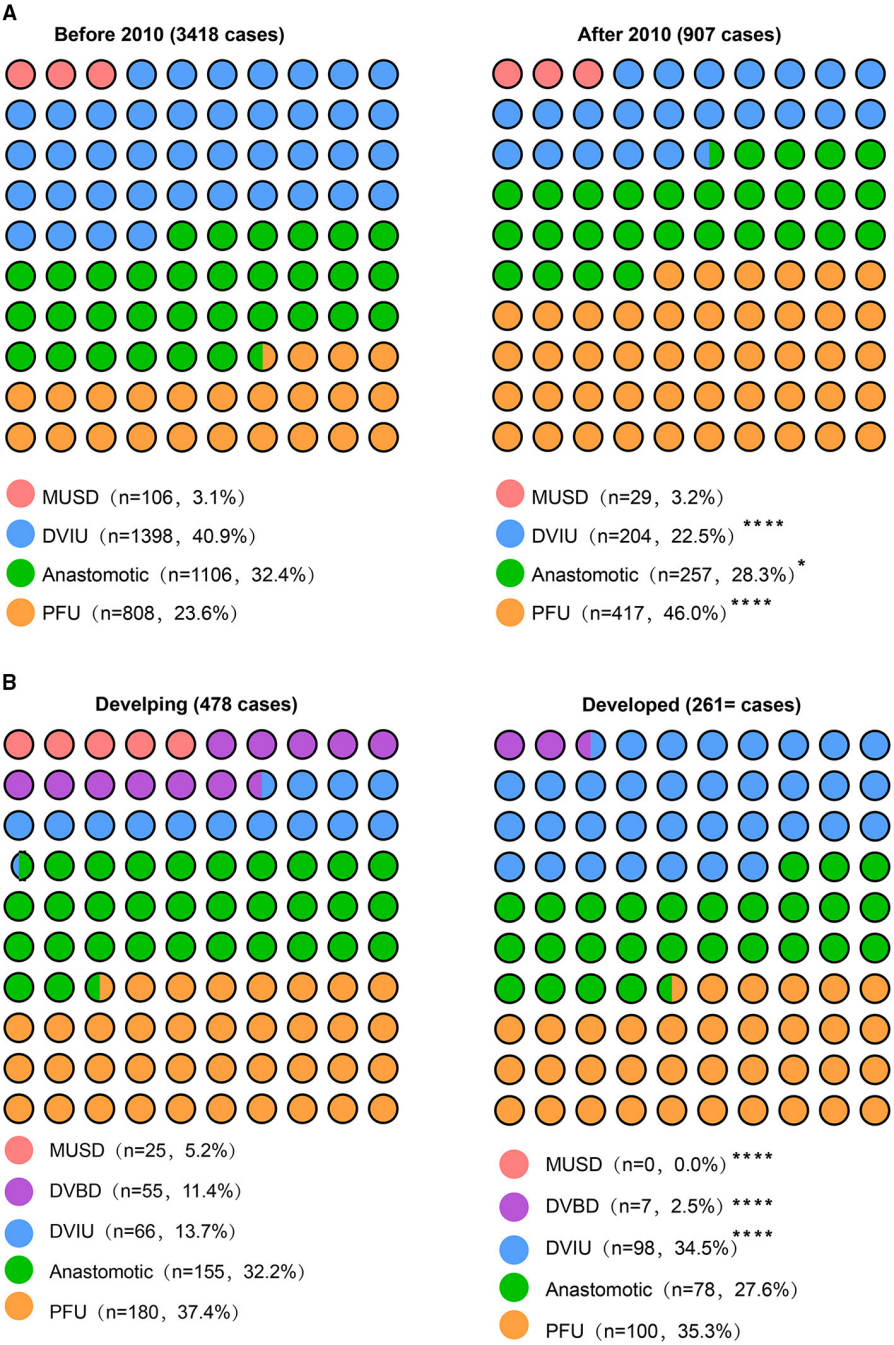
Treatment options between the two groups. **(A)** The patients were grouped by treatment time; **(B)** the patients were grouped by developing and developed regions. ^*^ = *p* < 0.05; ^**^ = *p* < 0.01; ^***^ = *p* < 0.001; ^****^ = *p* < 0.0001.

Clinical data of 11 centers in Mainland China extracted from five studies were included in the pooled analysis. A trend over time is shown in Figures 1A, 2A that urethral stricture happened more in the bulbar urethra and multiple sites after 2010 (18.4 vs. 14.8%; 9. vs. 5.4%, respectively), urethral stricture caused by trauma, hypospadias surgery, and urethra infection decreased (34.3 vs. 54.2%; 4.8 vs. 8.4%; 4.2 vs. 6.3%, respectively), while urethral stricture caused by the transurethral procedure, instillation of the bladder, and unknown causes increased after 2010 (30.5 vs. 17.1%; 3.2 vs. 2.%; 5.4 vs. 2.8%, respectively), treatment options for direct visual internal urethrotomy, and anastomotic urethroplasty decreased (22.5 vs. 40.9%; 28.3 vs. 32.4%, respectively), while pedicle flap urethroplasty increased (46 vs. 23.6%) after 2010.

A trend over the economic level is shown in Figures 1B, 2B, that urethral stricture happened more in the penile and bulbar urethra (30 vs. 22.4%; 27.2 vs. 18.8%, respectively), less in the urethral orifice and membranous urethra after 2010 (1.9 vs. 5.2%; 29.9 vs. 37.9%, respectively), urethral stricture caused by indwelling catheter, radiotherapy, and lichen sclerosus decreased (0 vs. 2.9%; 0 vs. 2.9%; 2.3 vs. 9.%, respectively), while urethral stricture caused by the transurethral procedure and unknown causes increased after 2010 (28.7 vs. 16.5%; 13.8 vs. 4.%, respectively), treatment options for mental urethral sound dilatation and direct visual balloon dilatation (0 vs. 5.2%; 2.5 vs. 11.4%, respectively), while direct visual internal urethrotomy increased (34.5 vs. 13.7%) after 2010. Overall, the results of the pooled analysis were partially consistent with the present study. Of note, the source of patients in large medical centers is complex to some degree; although it is a stable trend that people choose to visit a doctor in a near hospital for convenience and medical insurance policy, it might cause bias of result to divide the level of socioeconomic development by the location of the hospital.

## Discussion

With the rapid social-economic development in China, the etiology and treatment for MUS have changed in recent years. The present retrospective study revealed that trauma has always been the main cause of MUS in Hunan Province, which is much higher than in developed countries and is similar to that in developing countries (5–8). These have probably resulted from a large number of electric bicycles and industrial and mining enterprises in Hunan Province. The present study revealed the increasing MUS was caused by iatrogenic injuries, which can be treated through transurethral surgery, indwelling catheters, cystoscopy, prostatectomy, radiotherapy, and hypospadias surgery (9). Cystoscopy and transurethral resection of the prostate have been widely performed in all levels of hospitals in Hunan, which also lead to more iatrogenic injuries; a strict grasp of indications and contraindications are needed. Additionally, with the use of three-dimensional conformal radiotherapy technology in Hunan, complications after pelvic and perineal radiotherapy reduced (10).

The pooled analysis further showed the increase of MUS was caused by the transurethral procedure and the decrease of stricture was caused by urethra infection, because of the gradual promotion of transurethral minimally invasive surgery and improvement of sanitary conditions in Mainland China. In addition, the pooled analysis revealed that the MUS site was different with treatment time and regions, which might be related to the etiological changes of MUS. The pooled analysis of the two parts both reflected the trend of disease characteristics with the socioeconomic development from a larger scale, which thereby better strengthened some of the conclusions of the present study. However, the pooled analysis also showed some different or even competing results from this study. The positive reason may be the increase in statistical power by the large sample, and the negative reason may be the heterogeneity between studies, which led to bias.

With the improvement of surgical technology and growing demands for quality of life and reduction in complications, more and more patients with complex MUS received flap urethroplasty in our hospital, which was consistent with the results of pooled analysis. Urethroplasty is of long-term effectiveness and is being increasingly performed in developed countries (9). In China, the oral mucosa, foreskin flaps, bladder mucosa, colonic mucosa, and other tissue flaps have been used for urethroplasty (11, 12), while the main flaps used are foreskin flaps and scrotal flaps in our hospital. Additionally, treatments for simple MUS are also turning to endoscopic minimally invasive treatment or even urethroplasty.

The common causes for hematuria were lack of electric energy and urethral mucosa injury during the urethral dilatation. In Group B, the less use of blind metal sound urethral dilatation might contribute to reducing bleeding complications. Open surgery often led to penile curvature, abnormal ejaculation, and urethral fistula (13–15); the patients in Group B mainly received endoscopic surgery, which may be the reason for reducing those complications. Additionally, more complex MUS received pedicle flap urethroplasty rather than anastomotic urethroplasty, which might also contribute to less penile curvature.

However, there are some limitations to this study. First, the present study is a single-center investigation with a limited duration of follow-up. Second, the present study included the patients who failed to recover from multiple treatments in other hospitals, which might influence treatments. Third, the heterogeneity between studies might lead to bias in the pooled analysis.

## Conclusion

The present study still indicates the trends of MUS in Hunan, or even in Mainland China, which is socioeconomic development related. The main causes of urethral stricture in the past 10 years were still trauma and iatrogenic injuries. With the increase of intracavitary minimally invasive treatment and flap urethroplasty, the curative effect is increasing, while iatrogenic urethral stricture cannot be ignored.

## Data Availability Statement

The raw data supporting the conclusions of this article will be made available by the authors without undue reservation.

## Ethics Statement

The studies involving human participants were reviewed and approved by the Ethics Committee of the Second Xiangya Hospital of Central South University. The patients/participants provided their written informed consent to participate in this study.

## Author Contributions

YW and SP: conception and design. YW and SP: administrative support. XC, MD, LZ, YL, and SC: provision of study materials or patients. XC, MD, MP, LZ, YL, SP, and SC: collection and assembly of data. XC, MD, MP, and SP: data analysis and interpretation. XC and MD: manuscript writing. All authors: final approval of the manuscript.

## Funding

This work was supported by grants from the Hunan Province Innovation Platform and Talent Program-Clinical Medical Research Center (Grant No. 2019SK4003) and The Scientific Research Project of Hunan Health Commission (Grant Nos. C2019162 and B2019161).

## Conflict of Interest

The authors declare that the research was conducted in the absence of any commercial or financial relationships that could be construed as a potential conflict of interest.

## Publisher's Note

All claims expressed in this article are solely those of the authors and do not necessarily represent those of their affiliated organizations, or those of the publisher, the editors and the reviewers. Any product that may be evaluated in this article, or claim that may be made by its manufacturer, is not guaranteed or endorsed by the publisher.

## Abbreviations

MUS: male urethral stricture
IPSS: international prostate symptom score
QoL: quality of life score
Qmax: maximal urinary flow rate.

## References

[1] Benson CR Hoang L Clavell-Hernández J Wang R. Sexual dysfunction in urethral reconstruction: a review of the literature. Sex Med Rev. (2018) 6:492–503. 10.1016/j.sxmr.2017.09.002 29108977

[2] D'hulst P Floyd MS Jr Castiglione F Vander Eeckt K Joniau S Van der Aa F. Excision and primary anastomosis for bulbar urethral strictures improves functional outcomes and quality of life: a prospective analysis from a single centre. Biomed Res Int. (2019) 2019:7826085. 10.1155/2019/7826085 30809546 PMC6364126

[3] Xu YM Song LJ Wang KJ Lin J Sun G Yue ZJ. Changing trends in the causes and management of male urethral stricture disease in China: an observational descriptive study from13centres. BJU Int. (2015) 116:938–44. 10.1111/bju.12945 25294184

[4] Turner-Warwick R. Complex traumatic posterior urethral strictures. J Urol. (1977) 118:564–74. 10.1016/S0022-5347(17)58109-4 916051

[5] Agochukwu-Mmonu N Srirangapatanam S Cohen A Breyer B. Female urethral strictures: review of diagnosis, etiology, and management. CurrUrol Rep. (2019) 20:74. 10.1007/s11934-019-0933-1 31705324

[6] Astolfi RH Lebani BR Krebs RK Dias-Filho AC Bissoli J Cavalcanti AG. Specific characteristics of urethral strictures in a developing country (Brazil). World J Urol. (2019) 37:661–6. 10.1007/s00345-019-02696-9 30810832

[7] Ansari MS Yadav P Srivastava A Kapoor R Ashwin Shekar P. Etiology and characteristics of pediatric urethral strictures in a developing country in the 21st century. J Pediatr Urol. (2019) 15:403.e1–e8. 10.1016/j.jpurol.2019.05.020 31301979

[8] Cotter KJ Hahn AE Voelzke BB Myers JB Smith TG 3rd Elliott SP. Trends in urethral stricture disease etiology and urethroplasty technique from a multi-institutional surgical outcomes research group. Urology. (2019) 130:167–74. 10.1016/j.urology.2019.01.046 30880075

[9] Zhou SK Zhang J Sa YL Jin SB Xu YM Fu Q. Etiology and management of male iatrogenic urethral stricture: retrospective analysis of 172 cases in a single medical center. Urol Int. (2016) 97:386–91. 10.1159/000444592 27296973

[10] Zelefsky MJ Levin EJ Hunt M Yamada Y Shippy AM Jackson A. Incidence of late rectal and urinary toxicities after three-dimensional conformal radiotherapy and intensity-modulated radiotherapy for localized prostate cancer. Int J Radiat Oncol Biol Phys. (2008) 70:1124–9. 10.1016/j.ijrobp.2007.11.044 18313526

[11] Wang Z Zeng X Chen R Wang T Hu J Wang S. Free bladder mucosa graft harvested by water-jet: A novel, minimally invasive technique for urethral reconstruction. Exp Ther Med. (2018) 16:2251–6. 10.3892/etm.2018.6469 30186465 PMC6122421

[12] Xu YM Qiao Y Sa YL Zhang J Fu Q Song LJ. Urethral reconstruction using colonic mucosa graft for complex strictures. J Urol. (2009) 182:1040–3. 10.1016/j.juro.2009.05.030 19616803

[13] Favre GA Carminatti T Gil SA González IT Giudice CR. Safety and efficacy of urethroplasty based on age groups. ActasUrolEsp. (2020) 45:557–63. 10.1016/j.acuroe.2021.07.004 34526253

[14] Abrate A Gregori A Simonato A. Lingual mucosal graft urethroplasty 12 years later: Systematic review and meta-analysis. Asian J Urol. (2019) 6:230–41. 10.1016/j.ajur.2019.01.001 31297314 PMC6595159

[15] Saavedra AA Rourke KF. Characterization and outcomes of urethroplasty for hypospadias-associated urethral strictures in adults. Can Urol Assoc J. (2019) 13:E335–40. 10.5489/cuaj.5863 31039108 PMC6877363

